# Coping with salt stress-interaction of halotolerant bacteria in crop plants: A mini review

**DOI:** 10.3389/fmicb.2023.1077561

**Published:** 2023-02-02

**Authors:** Kesava Priyan Ramasamy, Lovely Mahawar

**Affiliations:** ^1^Department of Ecology and Environmental Science, Umeå University, Umeå, Sweden; ^2^Department of Plant Physiology, Faculty of Agrobiology and Food resources, Slovak University of Agriculture, Nitra, Slovakia

**Keywords:** salinity, halotolerant bacteria, crop plants, biotechnological applications, plant-microbe interaction

## Abstract

Salinity is one of the major environmental abiotic stress factors that limit the growth and yield of crop plants worldwide. It is crucial to understand the importance of several adaptive mechanisms in plants toward salt stress so as to increase agricultural productivity. Plant resilience toward salinity stress is improved by cohabiting with diverse microorganisms, especially bacteria. In the last few decades, increasing attention of researchers has focused on bacterial communities for promoting plant growth and fitness. The biotechnological applications of salt-tolerant plant growth-promoting rhizobacteria (PGPR) gained widespread interest for their numerous metabolites. This review provides novel insights into the importance of halotolerant (HT) bacteria associated with crop plants in enhancing plant tolerance toward salinity stress. Furthermore, the present review highlights several challenges of using HT-PGPR in the agricultural field and possible solutions to overcome those challenges for sustainable agriculture development in the future.

## 1. Introduction

Plants, due to their sessile nature, experience several environmental (abiotic and biotic) stresses during different developmental stages of their life cycle. The major abiotic stresses are drought, heavy metals, salinity, temperature, and ultraviolet (UV) light. Salinity is one of the extremely critical threats in the agricultural field, impacting one-fourth to one-third of crop production worldwide (Kumar et al., 2022). According to the Food and Agricultural Organization (FAO) report, over 424 million hectares (Mha) of topsoil (0–30 cm) (85% saline, 10% sodic, and 5% saline sodic) and 833 million hectares of subsoil (30–100 cm) (62% saline, 24% sodic, and 14% saline sodic) among 85% of the global land area are affected by salinity stress (Food Agriculture Organizations of the United Nations, 2022).

**Figure 1:**
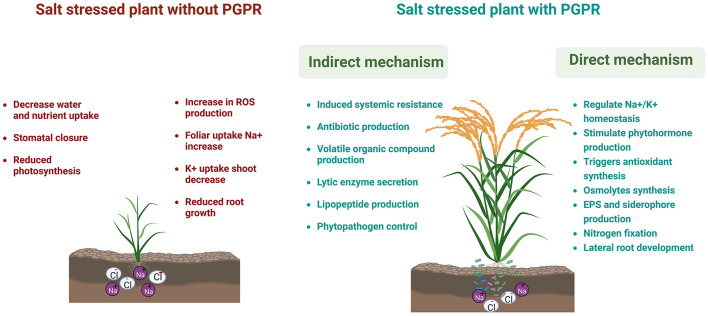
An underlying mechanism of halotolerant plant growth-promoting rhizobacteria (HT-PGPR) in alleviating salinity stress in crop plants. Licensed from biorender.

Soil salinity occurs mainly due to poor agricultural practices (high salt content water used for irrigation and fertilization) and the flow of saline water from the sea, rivers, etc., specifically in arid and semiarid regions (Zhang et al., 2021). Moreover, scarcity of rainfall and an increase in sea level due to climate change often cause the soil to become saline (Kumar et al., 2022). As a result, it produces hyperionic and hyperosmotic stresses in plant cells that impact the plant's growth (Kalaji et al., 2011) (Figure 1). Due to high osmotic stress, the uptake and transport of essential nutrients to a plant are affected highly (Farooq et al., 2015). Salinity stress affects the physiological development of plants (causes stomatal closure and premature senescence, reduces the rate of photosynthesis, and increases oxidative damage) (Mahawar and Shekhawat, 2019) and soil microbiota adversely, thus critically affecting complete soil health (Dubey et al., 2022) (Figure 1). However, plants must overcome salinity stress by modulating various morphophysiological and molecular responses (Zhao et al., 2020), such as improving the synthesis of phytohormones and osmoprotectants, upregulating antioxidant activities, and maintaining sodium ion (Na^+^) homeostasis and compartmentalization (Arif et al., 2020).

Another strategy to overcome salinity stress in plants is to cohabit with diverse halotolerant (HT) microorganisms that enhance growth, stress tolerance, and nutrient uptake in plants, thus restoring the crop yield (Etesami and Beattie, 2018; Etesami and Glick, 2020; Orhan, 2021). Halotolerant (HT) microorganisms that interact with plants are (1) rhizosphere, (2) epiphytes, and (3) endophytes (Andrews and Harris, 2000; Hardoim et al., 2015). The rhizosphere region in plants serves as natural hotspots (reservoirs) for various microorganisms, especially bacteria (Ling et al., 2022). One among such bacteria pertains to plant growth-promoting rhizobacteria (PGPR) that colonize the rhizosphere of the plant species. Many studies have proven that salt-tolerant plant growth-promoting rhizobacteria (PGPR) are supplied by various mechanisms to plants (Mishra et al., 2021). The important function of PGPR is to boost key physiological processes in plants, including photosynthesis, source–sink relationships, mineral and water uptake (Ilangumaran and Smith, 2017), fixation of atmospheric nitrogen, prevention of phytopathogens, improvement in the production of metabolites and phytohormones, such as indole-3-acetic acid (IAA), gibberellic acid (GA3), and cytokinin, solubilization of phosphate, and production of siderophores (Kumar and Verma, 2018). However, numerous studies demonstrated that different plant species colonize their own microbial communities (Kuske et al., 2002). Isolation and identification of the specific plant-based microorganism using microbiological and molecular methods would promote research on the plant–microbe interactions. In recent decades, the use of “omics” technologies, such as transcriptomics, proteomics, and metabolomics, to study the regulatory networks of halotolerant plant–bacteria interaction has increased.

The present review focuses on recent advances in plant–bacteria interactions and the underlying mechanisms of rhizosphere-residing bacterial species in a plant's response under salinity stress at the physiological and molecular levels. Moreover, the application and biotechnological potential of salt-tolerant PGPR in saline conditions have been explored. The present review also aimed to explore the major challenges of using PGPR in the agricultural field and their possible scientific solutions.

## 2. Plant growth-promoting rhizobacteria in crop's adaptation toward salinity stress

Plants adopted three main strategies to overcome sodium chloride (NaCl) stress and survive in the saline environment–osmotic stress tolerance, Na^+^/Cl^−^ exclusion, and tolerance to accumulate Na^+^/Cl^−^. Osmotic stress tolerance is mediated by a decrease in stomatal conductance, while the salinity stress-induced ionic response activates signal perception and transduction that limits the uptake, translocation, and accumulation of Na^+^ in the cell (Rahman et al., 2021). The plant adaptation toward salinity stress is improved by cohabiting with diverse saline soil microbes known as halotolerant plant growth-promoting rhizobacteria (HT-PGPR) that not only allow plants to persist in salt habitat but also improve their growth and soil-related properties (Hidri et al., 2022). In recent years, the importance of halotolerant plant growth-promoting rhizobacteria (HT-PGPR) in alleviating salinity stress in crop plants has increased. HT-PGPR improve the productivity of the saline-agroecosystem, either directly by producing several beneficial metabolites, such as exopolysaccharides, siderophores, volatile organic compounds (VOCs), compatible osmolytes, and phytohormones (Bhat et al., 2020), or indirectly by regulating the expression of stress-related genes and inhibiting the phytopathogen effects (Prasad et al., 2019) (Figure 1). Several halotolerant bacteria, including *Rhizobium, Arthrobacter, Flavobacterium, Alcaligenes, Pseudomonas*, and *Azospirillum*, were found to improve crop salinity tolerance (Saghafi et al., 2019a; Kumar Arora et al., 2020).

Halotolerant bacteria form biofilms and tend to produce extracellular polymeric substances/exopolysaccharides (EPSs) in a stressful environment (Haque et al., 2022). EPS production by HT-PGPR differs with the bacterial growth phase and external environment, such as nutrient medium composition, type of stress stimuli, pH, and temperature (Kumar Arora et al., 2020). Under a saline environment, EPS forms ~40–90% of the bacterial weight (Sunita et al., 2020). EPS forms a nutrient-rich sheath around the plant's roots called the rhizosheath (which also serves as a carbon source), which improves the availability and uptake of nutrients and water from the rhizosphere and acts as a physical barrier against ionic salts and phytopathogens (Mishra and Arora, 2018; Kumar Arora et al., 2020). Previously, Mahmood et al. (2016) studied the role of EPS-producing halotolerant *Enterobacter cloacae* and *Bacillus drentensis* in improving the growth of salt-stressed mung bean by increasing the water uptake and nutrient availability in crop plants. EPS is also associated with soil aggregation, humification, water retention, nodulation, quorum sensing, and the establishment of microbial diversity that protects plant cells from desiccation in a saline environment (Kumar Arora et al., 2020). Furthermore, EPS possesses antioxidant properties that confer tolerance against salinity-induced oxidative damage (Sunita et al., 2020). Inoculation of *Pantoea alhagi* NX-11, an EPS-producing endophyte, alleviates salt stress damage and improves the growth of *Oryza sativa* by stimulating antioxidant activity (Sun et al., 2020). In another study, the combined effect of silicon dioxide (SiO_2_) nanoparticles and exopolysaccharide (EPS)-producing bacterial inoculum on the upregulation of antioxidant activities in *Solanum lycopersicum* under salinity stress has been reported (Isfahani et al., 2019). Moreover, HT-PGPR in a stress environment produce low-molecular-weight, lipophilic metabolites known as volatile organic compounds (VOCs) (Sunita et al., 2020). The VOCs promote the growth and adaptability of stressed plants by stimulating the synthesis of siderophores, osmoprotectants, and phytohormones, triggering the expression of HKT1/K^+^ transporters and regulating virulence factors and bacterial motility in the plant–microbe interaction (Bhat et al., 2020). The upregulation of HKT antiporters by VOC-producing HT-PGPR strains, such as *Dietzia natronolimnaea* (Bharti et al., 2016), *Arthrobacter nitroguajacolicus* (Safdarian et al., 2019) in *Triticum aestivum*, and *Bacillus amyloliquefaciens* SQR9 (Chen et al., 2016) in *Zea mays*, has been studied under salinity stress. Another major challenge for salinity-stressed crops is the low availability of soluble ferrous ions (Fe^2+^) (iron form uptake by plants), which is determined primarily by the soil pH (available at acidic pH 6). Saline soils have alkaline pH (pH > 6.5) that causes the oxidation of ferrous ion (Fe^2+^) to ferric ion (Fe^3+^) and reduces the availability of iron to plants (Mahawar et al., 2022). The production of siderophores by HT-PGPR chelates Fe^3+^ and compensates the iron (Fe) requirement in salt-stressed crops (Kumar Arora et al., 2020). In this context, Mukherjee et al. (2019) studied the role of siderophores produced by the HT-PGPR strain, *Halomonas* sp., in promoting the growth and productivity of *Oryza sativa* growing in a saline environment.

Synthesis and accumulation of osmolytes/osmoprotectants are one of the earliest responses of plants to combat osmotic and oxidative damage imposed by salinity stress (Jogawat, 2019). HT-PGPR help plants to accumulate compatible osmolytes (amino acids, soluble sugars, and polyols) in a saline environment to minimize osmotic stress, maintain high turgor pressure, and sustain ion equilibrium in the cytoplasm. Moreover, HT-PGPR have been reported to upregulate osmolyte biosynthesis genes (mainly proline) and control stomatal conductance and transpiration rate (Saghafi et al., 2019b) to mitigate water stress in plants (Sunita et al., 2020). Bioinoculation of a halotolerant PGPR, *Bacillus fortis* SSB21, in capsicum, improved proline synthesis and expression of stress-related genes, namely, the pepper pathogen-induced protein gene (CAPIP2), a putative ketoacyl-ACP reductase (CaKR1), pepper osmotin-like protein 1 (CaOSM1), and pepper class II basic chitinase (CAChi2) during salinity stress (Yasin et al., 2018). Similarly, the inoculation of *Paenibacillus yonginensis* DCY84^T^ into *Panax ginseng* seeds subjected to salinity stress increases polyamine, total soluble sugar, chlorophyll and proline content, abscisic acid (ABA) synthesis, and the upregulation of stress-responsive genes in stressed plants (Sukweenadhi et al., 2018).

The modulation of phytohormone synthesis against stress environment is another important characteristic of HT-PGPR to confer symbiotic association and promote the growth and productivity of stressed plants (Kumar Arora et al., 2020). Recent studies reported that, under saline condition, phytohormone synthesis genes, mainly IAA, are upregulated in salt-tolerant PGPR that compensate for growth hormones' requirement in plants, alter plant root's morphology, and exclude excess ionic salts (Bhat et al., 2020). Several *in vitro* studies revealed that the improved IAA production by HT-PGPR in plants reduces tap root growth, promotes the elongation of root hairs, and increases the number and length of lateral roots. Thus, it improves crop growth by increasing the availability and uptake of water and nutrients (Nawaz et al., 2020; Grover et al., 2021; Sarker et al., 2022). Inoculation of *Pseudomonas putida, Pseudomonas stutzeri*, and *Stenotrophomonas maltophilia* in *Coleus forskohlii* enhanced the production of IAA, gibberellic acid, and cytokinin in plants (Patel and Saraf, 2017). Similarly, *Pseudomonas* sp. enhanced the production of gibberellins in *Glycine max* (Kang et al., 2014) and cytokinin in *Zea mays* (Sandhya et al., 2010) growing under sodium chloride (NaCl) stress. Apart from growth hormones, PGPR are capable of synthesizing and modulating the gene expression of stress hormones (abscisic acid (ABA) and ethylene) (Bhat et al., 2020). A study conducted by Bharti et al. (2016) demonstrated the role of halotolerant strain *Dietzia natronolimnaea* STR1 in upregulating the expression of ABA signaling cascade genes, such as ABA response elements (TaABARE) and 12-oxophytodienoate reductase 1 (TaOPR1), which stimulates the expression of the salt stress-induced gene, *TaST*, in *Triticum aestivum*. Ethylene, another stress hormone, promotes plant tolerance toward salinity stress but constrains their growth and productivity. HT-PGPR secrete 1-aminocyclopropane-1-carboxylase (ACC) deaminase that metabolizes ACC (ethylene precursor) into α-ketoglutarate and ammonia, thus hampering ethylene synthesis in plants (Bhat et al., 2020). Panwar et al. (2016) studied that ACC deaminase-producing strains of *Enterobacter* spp. and *Pseudomonas fluorescens* increased the growth and yield of *Zea mays* in a saline environment.

In addition to producing plant-beneficial metabolites, HT-PGPRs constrict the influx of Na^+^ by regulating the Na^+^/K^+^ homeostasis and modulating the expression of different salt-tolerant genes, such as salt overly sensitive (SOS), high-affinity K^+^ transporters (HKT), Na^+^/H^+^ antiporter (NHX), aquaporins (AQPs), and antioxidants, thereby conferring plants resistance toward salinity stress. The treatment of HT-PGPR *Bacillus subtilis* (GB03) reduces Na^+^ uptake in the halophyte grass *Puccinellia tenuiflora* by the upregulation of PtHKT1;5 and PtSOS1 and the downregulation of the PtHKT2;1 gene (Niu et al., 2016). A similar mechanism of *Bacillus subtilis* (GB03) was studied in *Triticum aestivum* growing under a saline environment (Zhang et al., 2014). Long-term exposure to a saline environment causes water deficiency in crops. Inoculation of HT-PGPR *Bacillus megaterium* upregulates the expression of the aquaporin genes ZmPIP1-1 and PIP2 in *Zea mays* that increase the water uptake in salt-stressed plants (Marulanda et al., 2010). Additionally, HT-PGPR augment salt tolerance in host plants by modulating the expression and activity of antioxidants (Kumar Arora et al., 2020). The priming of *Panax ginseng* seeds with salt-tolerant *Paenibacillus yonginensis* DCY84T improved the expression of *PgAPX* and *PgCAT* genes in salt-stressed plants (Sukweenadhi et al., 2018). In another study, HT-PGPR *Bradyrhizobium* and *Pseudomonas graminis* during a saline environment triggered the accumulation of ascorbate and glutathione in *Vigna unguiculata* (Santos et al., 2018). Similarly, inoculation of *B. megaterium* UPMR2 and *Enterobacter* sp. UPMR18 in *Abelmoschus esculentus* upregulates the expression of stress-related genes such as superoxide dismutase (SOD), ascorbate peroxidase (APX), catalase (CAT), glutathione reductase (GR), and dehydroascorbate reductase (DHAR) under NaCl stress (Habib et al., 2016). Supplementary Table 1 shows the list of bacterial species and their mechanisms in plant hosts to alleviate salinity stress.

## 3. The major challenges of HT-PGPRs in field conditions and their probable scientific solutions

**Figure 2:**
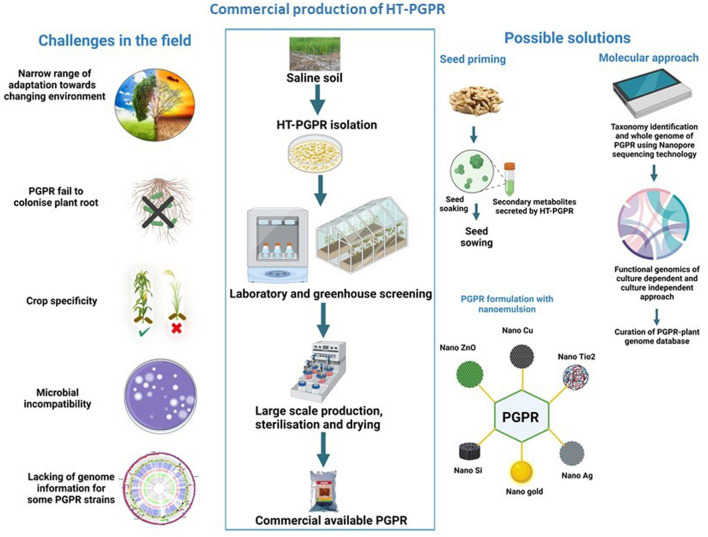
The major challenges of using halotolerant plant growth-promoting rhizobacteria (HT-PGPR) in the agricultural field and possible solutions to overcome them. The square box represents the steps involved in the commercial production of PGPR under controlled condition, while the **(left)** and **(right)** sides of the square explain the various problems involved in employing PGPR in the field and probable solutions to conquer them. Licensed from biorender.

The HT-PGPR is commercially used in agriculture due to its several advantages over synthetic agrochemicals in stimulating the growth and yield of economically important crops in both normal and stress conditions. Several PGPR-based bioformulations and products are available on the market and many of them are still in the development process. PGPR production from laboratory to field is a complex process that is completely based on laboratory screening assays and field trials (Figure 2) (Backer et al., 2018). However, the commercial PGPR inoculants did not show similar promising effects in promoting crop growth in agricultural fields as those under controlled laboratory conditions. The major challenge in reducing the PGPR performance in field conditions is climate change. Considerable climate change not only impacts plant physiology but also affects the diversity, abundance, colonization, and activities of plant-associated microbial communities (Tabassum et al., 2017). Moreover, the climate in different ecological zones also affects the PGPR efficiency, as there is no potent commercial inoculant that has a similar response in all ecological zones (Liu et al., 2022). For an effective PGPR inoculum, the inoculated strains must colonize the plant roots and propagate into the rhizosphere. Certain bacterial strains fail to colonize the roots and are thus incompetent to promote plant growth in the field, as that under controlled conditions (Figure 2). Crop variety and PGPR strain are other important aspects that must be considered while using bioinoculants. The impact of specific PGPR strains or consortia on crop growth and yield varies with the crop cultivars (Figure 2). Furthermore, inoculation of defined microbial strain depends on crop necessity (pathogen resistance, stress tolerance, yield stimulation, etc.) (Tabassum et al., 2017). The carrier selection also plays a crucial role in PGPR performance. An inappropriate carrier reduces the survival and efficiency of bacteria in the rhizosphere. Moreover, the PGPR performance in a carrier material differs from strain to strain (Sohaib et al., 2020). The compatibility of microbes in consortia is another factor. Many bacterial species have antagonistic interactions with other strains, reducing PGPR efficiency in the field (Tabassum et al., 2017). Environmental safety associated with PGPR strain is an additional concern that cannot be neglected. Microbial inoculation triggers substantial changes at the level of native non-targeted microbial communities. Consortia/PGPR inoculants compete with the indigenous soil microbial population (for nutrients, habitat, trading metabolites, etc.) that results in changes to the community structure, loss of native diversity, and a rise in alien host diversity (Figure 2) (Thakur et al., 2019). Thus, all these aforementioned factors reshape the functionality of resident soil communities by provoking secondary succession (Liu et al., 2022).

The PGPR efficiency is dependent directly on soil properties, plant signaling molecules, and the surrounding environment. A careful selection of multipotent environment-friendly PGPR strains that can withstand a wide range of environments is the key to deploying a sustainable approach in crop improvement to the changing environment. In-depth studies on laboratory screening procedures are required for selecting suitable eco-friendly bioinoculants that favor the growth of crops under saline conditions (Figure 2). Recent advancements in “rhizosphere engineering” could mitigate salt stress by engineering the rhizosphere microbiome. For example, genome editing technology, such as Clustered Regularly Interspaced Short Palindromic Repeats (CRISPR)/CRISPR-associated protein 9 (Cas9), is a fast, eco-friendly, and effective way to understand the plant–PGPR interactions, in particular, to target pathways involved in various metabolites (Prabhukarthikeyan et al., 2020). Another approach is to unravel the “blackbox” of PGPRs using next-generation sequencing (NGS) to explore microbial diversity under salinity stress. In recent years, the culture-independent approach has been increased to configure the bacteriobiome complex associated with various plant species. Recently, Poncheewin et al. (2022) studied the plant-associated lifestyle of *Pseudomonas* strains using genome properties (GPs) of common biological pathways' annotation system and the machine learning (ML) approach to differentiate functional features. However, the 16S ribosomal RNA (16S rRNA) profiling of bacterial communities has a few limitations (such as the primer design, coverage, sequencing errors, and pipeline analysis from different sequencing platforms). To resolve these challenges in a culture-independent approach, the following strategies need to be included: (1) optimization of primer pairs, (2) sequencing depth and coverage, and (3) curated reference database. In addition, there is a need to develop a database to expand the knowledge of HT-PGPR—plant interaction for sustainable agriculture. In recent years, the increase in whole genome sequencing of PGPR and data availability has facilitated comparative genomics between host plants and endophytes. Moreover, the genome insights provide novel information about salinity-tolerant genes associated with specific interactions between host plants and PGPR. Seed priming with PGPR's secreted secondary metabolites is the other indirect approach to enhance crop productivity under salinity stress. The priming of seeds not only stimulates crop growth and yield in changing environments but also protects native soil microbial communities from direct exposure to PGPR. Moreover, the utilization of a nanoemulsion-based delivery system can improve PGPR performance. Nanoemulsion carriers can make PGPR more efficient in the agricultural field, due to their increase in solubility, stability, targeted delivery, controlled release, and cost-effectiveness (Ravichandran et al., 2022) (Figure 2).

## 4. Conclusion

Halotolerant plant growth-promoting rhizobacteria (HT-PGPR) are an excellent green alternative that facilitates crop plants to cope with increasing salinity stress. In recent years, the beneficial impact of PGPR on agriculture to yield economically important crops has increased. HT-PGPR promote crop production by several mechanisms (physiological and molecular level) in a saline environment. However, many molecular functions and signaling pathways of HT-PGPR used for promoting crop growth during the plant–microbe interaction are still unknown and need to be characterized. In addition, maintaining a similar efficiency of HT-PGPR in an agricultural field as that under controlled laboratory conditions is the other major field that needs to be focused on to accomplish sustainable agriculture. Isolation and genome sequencing of many unexplored novel PGPR strains could expand our knowledge by acquiring a better understanding of the PGPR-plant interaction with halophytes and for selecting specific multipotent broad ranges of strains or consortia. Moreover, biotechnological tools, such as rhizosphere engineering, next-generation sequencing, and a culture-independent approach, can be used to explore unidentified microbes in the complex bacteriobiome associated with plant species.

## Author contributions

KPR and LM conceptualized the idea and constructed the figures and table. Both authors contributed equally to the writing of this review and approved the final version.
