# Supplementary material for: Coping with salt stress-interaction of halotolerant bacteria in crop plants: A mini review
Source: Front Microbiol. 2023 Feb 2;14:1077561. doi: 10.3389/fmicb.2023.1077561 (PMC9932039; doi:10.3389/fmicb.2023.1077561)
Supplement: Supplementary file 1 [file Table_1.docx]

**Supplementary Table 1: PGPR role in alleviating salinity stress in crop plants by different mechanisms and the information on their genome availability based on NCBI database.**

| **Plant host** | **Bacteria** | **Functions** | **Whole Genome availability** | **References** |
| --- | --- | --- | --- | --- |
| *Oryza sativa* L. | *Anabaena oryzae*,  *Anabaena doliolum* | Accumulation of phenolic acids, phytohormones | - | Singh et al., 2011; Zhang et al., 2017 |
|  | *Luteovulum sphaeroides* | Enhance available phosphorus | + | Khuong et al., 2021 |
|  | *Brevibacterium sediminis* | Growth promotion of rice seedling under salinity stress | + | Mahmud-Ur-Rahman et al., 2022 |
|  | *Methylobacterium oryzae* CBMB20 | Improves photosynthesis and reduces stress volatile emissions | + | Chatterjee et al., 2019 |
|  | *Brevibacterium linens* | Increases the accumulation of proline and glycine betaine | + | Ahmed et al., 2021 |
|  | *Streptomyces albidoflavus* | Increases proline content and sugar | + | Niu et al., 2022 |
|  | *Bacillus amyloliquefaciens* | Increases biomass, relative water content, proline and total soluble sugar | + | Chauhan et al., 2019 |
|  | *Enterobacter sp.* PR14 | Production of aminocyclopropane-1-carboxylate deaminase (ACCD) and antioxidant enzyme under salinity stress | - | Sagar et al., 2020 |
|  | *Bacillus pumilus* strain JPVS11 | Promotes photosynthetic pigments, proline and antioxidant production. | - | Kumar et al., 2021 |
|  | *Pantoea ananatis* D1 | phosphate solubilization, production of indole-3-acetic acid, 1-aminocyclopropane-1-carboxylic acid (ACC) deaminase and siderophore | - | Lu et al., 2021 |
|  | *Staphylococcus sciuri* ET101 | Increased growth rate and stimulated root growth | - | Taj and Challabathula, 2021 |
| *Triticum aestivum* L | *Bacillus altitudinis* WR10 | Improves wheat seed relative germination rate | - | Yue et al., 2019 |
|  | *Enterobacter cloacae* ZNP-3 | Indole-3-acetic acid (IAA) production, mineral phosphate solubilization, hydrogen cyanide (HCN) and ammonia production | - | Singh et al., 2017 |
|  | *Serratia marcescens* CDP-13 | Against fungal pathogen, IAA production, siderophore production, phosphate solubilization. | - | Singh and Jha, 2016 |
|  | *Azospirillum* *lipoferum* JA4∷*ngfp*15 *(*gfp-tagged) | Reduces salinity stress | - | Bacilio et al., 2004 |
|  | *Klebsiella* sp*.* SBP-8 | Possess 1-aminocyclopropane-1-carboxylatedeaminase (ACCD) activity | - | Singh et al., 2015 |
|  | *Pseudomonas* sp. JG7 and *Bacillus* sp. JG3 | Plant growth promotion | - | Vimal et al., 2018 |
| *Zea mays* | *Pseudomonas* sp*.* P8 | Inhibit phytopathogenic fungi, promotes growth and productivity | - | Peng et al., 2021 |
|  | *Peribacillus* sp. P10 |  | - |  |
|  | *Streptomyces* sp. X52 |  |  |  |

**References**

Ahmed, S., Heo, T. Y., Roy Choudhury, A., et al. (2021). Accumulation of compatible solutes in rice (*Oryza sativa L*.) cultivars by inoculation of endophytic plant growth promoting bacteria to alleviate salt stress. *Appl. Biol. Chem.* 64, 68. https://doi.org/10.1186/s13765-021-00638-x.

Bacilio, M., Rodriguez, H., Moreno, M., Hernandez, J-P., and Bashan, Y. (2004). Mitigation of salt stress in wheat seedlings by a gfp-tagged *Azospirillum lipoferum*. *Biol. Fertil. Soils* 40, 188-193. doi:10.1007/s00374-004-0757-z.

Chatterjee, P., Kanagendran, A., Samaddar, S., Pazouki, L., Sa, T-M., and Niinemets, Ü. (2019). *Methylobacterium oryzae* CBMB20 influences photosynthetic traits, volatile emission and ethylene metabolism in *Oryza sativa* genotypes grown in salt stress conditions. *Planta*, 249(6), 1903-1919. doi: 10.1007/s00425-019-03139-w.

Chauhan, P.S., Lata, C., Tiwari, S. et al. (2019). Transcriptional alterations reveal *Bacillus amyloliquefaciens*-rice cooperation under salt stress. *Sci Rep* 9, 11912. https://doi.org/10.1038/s41598-019-48309-8

Khuong, N., Huu, T., Thuc, L., Thu, L., Xuan, D., Quang, L., Nhan, T., Tran, H., Tien, P., Xuan, L. et al. (2021). Two strains of *Luteovulum sphaeroides* (purple nonsulfur bacteria) promote rice cultivation in saline soils by increasing available phosphorus. *Rhizosphere* 20, 100456. doi:10.1016/j.rhisph.2021.100456.

Kumar, A., Singh, S., Mukherjee, A., Rastogi, R. P., and Verma, J. P. (2021). Salt-tolerant plant growth-promoting *Bacillus pumilus* strain JPVS11 to enhance plant growth attributes of rice and improve soil health under salinity stress. *Microbiol. Res.* 242, 126616. doi: 10.1016/j.micres.2020.126616.

Lu, L., Chang, M., Han, X., Wang, Q., Wang, J., Yang, H., Guan, Q., and Dai, S. (2021). Beneficial effects of endophytic *Pantoea ananatis* with ability to promote rice growth under saline stress. *J. App. Microbiol.* 131(4), 1919-1931. https://doi.org/10.1111/jam.15082.

Mahmud-Ur-Rahman, Naser, I. B., Mahmud, N. U., Sarker, A., Hoque, M. N., and Islam, T. A. (2022). A Highly Salt-Tolerant Bacterium *Brevibacterium sediminis* Promotes the Growth of Rice (*Oryza sativa* L.) Seedlings. *Stresses*, 2, 275-289. https://doi.org/10.3390/ stresses2030020.

Niu, S., Gao, Y., Zi, H., Liu, Y., Liu, X., Xiong, X., et al. (2022). The osmolyte-producing endophyte *Streptomyces albidoflavus* OsiLf-2 induces drought and salt tolerance in rice via a multi-level mechanism. *Crop J.* 10(2), 375-386.

Peng, J., Ma, J., Wei, X., Zhang, C., Jia, N., Wang, X., et al. (2021). Accumulation of beneficial bacteria in the rhizosphere of maize (*Zea mays* L.) grown in a saline soil in responding to a consortium of plant growth promoting rhizobacteria. *Ann. Microbiol.* 71, 40. doi:10.1186/s13213-021-01650-8.d.

Sagar, A., Sayyed, R. Z., Ramteke, P. W., et al. (2020). ACC deaminase and antioxidant enzymes producing halophilic *Enterobacter* sp. PR14 promotes the growth of rice and millets under salinity stress. *Physiol. Mol. Biol. Plant.* 26, 1847-1854.

Singh, R. P., and Jha, P. N. (2016). The Multifarious PGPR Serratia marcescens CDP-13 Augments Induced Systemic Resistance and Enhanced Salinity Tolerance of Wheat (*Triticum aestivum* L.). *PLOS ONE* 11 (6), e0155026. doi:10.1371/journal.pone.0155026.

Singh, D., Prabha, R., Yandigeri, M., and Arora, D. (2011). Cyanobacteria-mediated phenylpropanoids and phytohormones in rice (*Oryza sativa*) enhance plant growth and stress tolerance. *Antonie van Leeuwenhoek* 100 (4), 557-568. doi:10.1007/s10482-011-9611-0.

Singh, R. P., Jha, P., and Jha, P. N. (2015). The plant-growth-promoting bacterium *Klebsiella* sp. SBP-8 confers induced systemic tolerance in wheat (*Triticum aestivum*) under salt stress. *J. Plant Physiol.* 184, 57–67. doi:10.1016/j.jplph.2015.07.002.

Singh, R. P., Jha, P., and Jha, P. N. (2017). Bio-inoculation of Plant Growth-promoting Rhizobacterium *Enterobacter cloacae* ZNP-3 Increased Resistance Against Salt and Temperature Stresses in Wheat Plant (*Triticum aestivum* L.). *J. Plant Growth Regul.* 36, 783-798. doi:10.1007/s00344-017-9683-9.

Taj, Z., and Challabathula, D. (2021). Protection of Photosynthesis by Halotolerant *Staphylococcus sciuri* ET101 in Tomato (*Lycoperiscon esculentum*) and Rice (*Oryza sativa*) Plants During Salinity Stress: Possible Interplay Between Carboxylation and Oxygenation in Stress Mitigation. *Front. Microbiol.* 11, 547750. doi:10.3389/fmicb.2020.547750.

Vimal, S. R., Gupta, J., and Singh, J. S. (2018). Effect of salt tolerant Bacillus sp. and Pseudomonas sp. on wheat (Triticum aestivum L.) growth under soil salinity: A comparative study. *Microbiol. Res.* 9 (1), 7462 doi:10.4081/mr.2018.7462.

Yue, Z., Shen, Y., Chen, Y., Liang, A., Chu, C., Chen, C., and Sun, Z. (2019). Microbiological Insights into the Stress-Alleviating Property of an Endophytic *Bacillus altitudinis* WR10 in Wheat under Low-Phosphorus and High-Salinity Stresses. *Microorganisms* 7(11), 508. doi:10.3390/microorganisms7110508.

Zhang, X., Zhang, R., Gao, J., Wang, X., Fan, F., Ma, X., et al. (2017). Thirty-one years of rice-green manure rotations shape the rhizosphere microbial community and enrich beneficial bacteria. *Soil Biol. Biochem.* 104, 208–217. doi: 10.1016/j.soilbio.2016.10.023.
